# UV sensing using film bulk acoustic resonators based on Au/n-ZnO/piezoelectric-ZnO/Al structure

**DOI:** 10.1038/srep09123

**Published:** 2015-03-16

**Authors:** Xiaolei Bian, Hao Jin, Xiaozhi Wang, Shurong Dong, Guohao Chen, J. K. Luo, M. Jamal Deen, Bensheng Qi

**Affiliations:** 1 Dept. of Info. Sci. & Electr. Eng., Zhejiang University and Cyrus Tang Center for Sensor Materials and Applications, 38 Zheda Road, Hangzhou 310027, China; 2 Institute of Renewable Energy & Environ. Technol., Univ of Bolton, Bolton, BL3 5AB, UK; 3 College of Internet of Things Engineering, Hohai University, Changzhou, 213022, China; 4 Electrical and Computer Engineering, McMaster University, Hamilton, ON L8S 4K1, Canada and ECE Department, Hong Kong University of Science and Technology, Kowloon, Hong Kong

## Abstract

A new type of ultraviolet (UV) light sensor based on film bulk acoustic wave resonator (FBAR) is proposed. The new sensor uses gold and a thin n-type ZnO layer deposited on the top of piezoelectric layer of FBAR to form a Schottky barrier. The Schottky barrier's capacitance can be changed with UV light, resulting in an enhanced shift in the entire FBAR's resonant frequency. The fabricated UV sensor has a 50 nm thick n-ZnO semiconductor layer with a carrier concentration of ~ 10^17^ cm^−3^. A large frequency downshift is observed when UV light irradiates the FBAR. With 365 nm UV light of intensity 1.7 mW/cm^2^, the FBAR with n-ZnO/Au Schottky diode has 250 kHz frequency downshift, much larger than the 60 kHz frequency downshift in a conventional FBAR without the n-ZnO layer. The shift in the new FBAR's resonant frequency is due to the junction formed between Au and n-ZnO semiconductor and its properties changes with UV light. The experimental results are in agreement with the theoretical analysis using an equivalent circuit model of the new FBAR structure.

Detection of ultraviolet (UV) radiation is very important in a number of areas such as UV radiation dosimetry, space-to-space communications, flame detection, UV astronomy, chemical/biological battle field reagent detectors, water purification, early missile threat warning, medicine, furnace control, and pollution monitoring^1^,^2^ There are various types of UV sensors based on ZnO materials^3^,^4^. Film bulk acoustic wave resonator (FBAR) is a good choice for future UV sensing application owing to its high sensitivity and possibility of integration with CMOS technology. FBAR devices have been widely used in wireless systems^5^,^6^,^7^,^8^ as filters^9^, oscillators^10^ and duplexers with better performance compared with surface acoustic wave or Quartz crystal devices.

FBAR sensors have excellent performance such as high sensitivity due to its high operating frequency and very high quality (Q) factor. For example, FBAR devices have been used as gravimetrical sensors to sense mass changes with a detection limit about ~ 10^−15^ g^11^. FBAR devices for simultaneous sensing of temperature and pressure have shown very high sensitivities of −17.4 and −6.1 ppm.kPa^−1^ 12. Also, FBAR-based biosensors are receiving more attentions due to the ever-increasing demands in healthcare applications^13^,^14^. To date, there are few studies reporting on UV sensing based on FBAR devices^15^. However, we believe that FBAR-based UV sensors with their excellent performance may increase their use. Therefore, in this work, we studied two types of UV sensors using different FBAR device structures and will show that they possess excellent performance characteristics.

## Results

### UV sensors based on two FBAR structures

**Figure 1:**
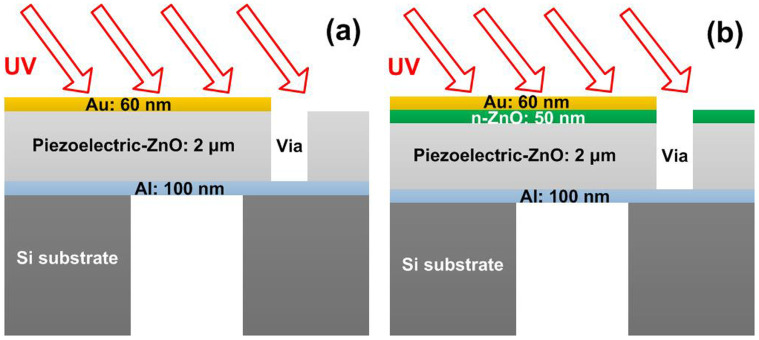
Schematics of (a) traditional FBAR device with back cavity, and (b) newly proposed FBAR device incorporating an n-ZnO semiconductor layer.

**Figure 2:**
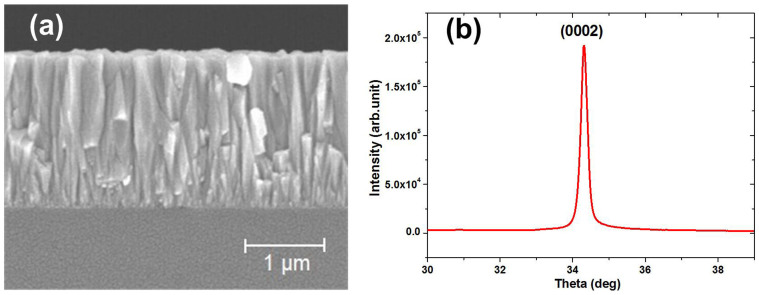
(a) SEM image of a cross-section of the ZnO layer, and (b) XRD rocking curve of the piezoelectric ZnO layer.

The traditional type of FBAR device is shown in Figure 1a. It is composed of a top electrode, a piezoelectric layer, a bottom electrode and an acoustic wave reflector. Air is an ideal acoustic wave reflector, so that an air cavity is formed under the bottom electrode. A via through the ZnO piezoelectric layer is used to connect the bottom electrode. Figure 2a shows a cross sectional scanning electron microscopy (SEM) image of the ZnO layer, and Figure 2b, the corresponding X-ray diffraction (XRD) rocking curve. The SEM and XRD results indicate that the ZnO layer consists of columnar structure with (0002) crystal orientation. The full-width half-maximum (FWHM) of the rocking curve of the (0002) peak is very narrow, about 0.22°, indicating the small angular dispersion of the crystallites around the c-axis and excellent ZnO crystal formation^12^.

UV light sensors based on Schottky diodes, with rapid response to UV light and low noise characteristics, have been widely reported^16^,^17^. Here, we propose a new type of FBAR by introducing a Schottky diode into the sensor as shown in Figure 1b. In this structure, a 50 nm thick n-type ZnO (n-ZnO) semiconducting film was introduced between the ZnO piezoelectric layer and the top gold (Au) electrode. The electron concentration measured using the Hall Effect was ~ 10^17^ cm^−3^. The top Au electrode and n-ZnO form a Schottky barrier^18^. The Schottky barrier's capacitance, which can be changed under UV exposure, in-turn, modifies the entire FBAR resonant frequency. The composite device makes use of the advantage of FBAR's very high sensitivity and Schottky diode's high response speed to UV light with low noise.

### The resonant frequency response to UV exposure

**Figure 3:**
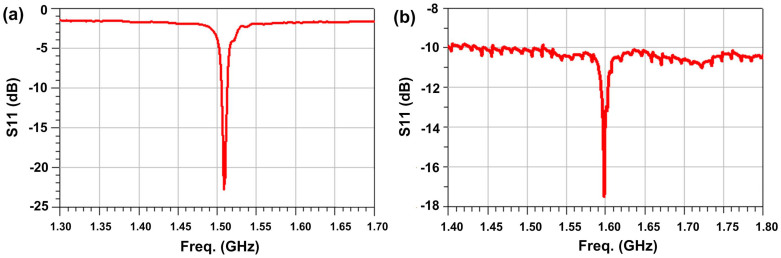
Frequency response of (a) a traditional FBAR device, and (b) a new FBAR device with an n-ZnO semiconductor layer.

The traditional type of FBAR has a resonant frequency of 1.5 GHz, and the newly proposed FBAR has a resonant frequency of 1.6 GHz. The Q-factor was calculated by −3 dB method, while the temperature coefficient of frequency (TCF) for the FBARs can be calculated by using the following equation,


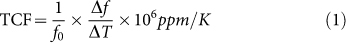
where *f_0_*, Δ*f*, Δ*T* are the fundamental frequency, frequency shift, temperature variation, respectively. The traditional FBAR has a TCF of 60–70 ppm/K and a Q-factor of ~ 600, while the new type of FBAR has a TCF of 25–35 ppm/K and a Q-factor of ~ 500. Figure 3 shows the reflection spectra, S11, of the traditional FBAR and the proposed new FBAR. The new type FBAR has a slightly lower Q-factor than that of the traditional structure FBARs. This means that the introduction of the n-ZnO layer has little influence on FBAR Q-factor and insertion loss, while it improves the FBAR's TCF.

**Figure 4:**
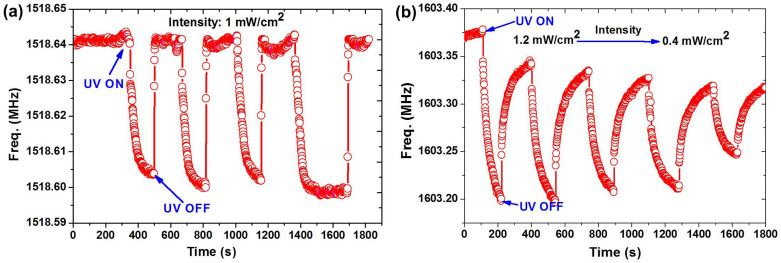
Frequency response of (a) a traditional FBAR under UV light with a fixed light intensity of 1 mW/cm^2^ and varying illumination time; and (b) the newly proposed FBAR under UV light with decreasing light intensity from 1.2 to 0.4 mW/cm^2^ at an interval of 0.2 mW/cm^2^.

**Figure 5:**
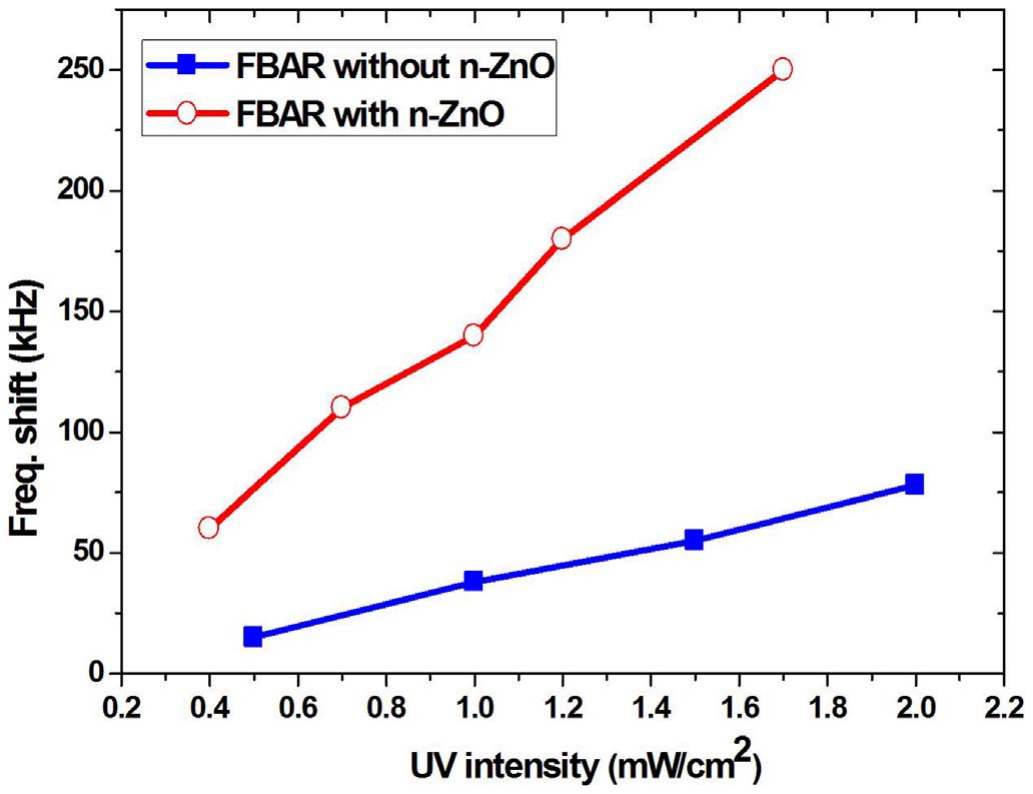
Comparison of frequency responses between the traditional FBAR and the new FBAR with an n-ZnO layer.

The experimental results of the resonant frequency response to UV exposure are shown in Figure 4. Under exposure of 365 nm UV light of an intensity of 1 mW/cm^2^, the proposed FBAR has a resonant frequency downshift of 140 kHz, compared with 40.0 kHz shift for the traditional FBAR. Comparing the proposed FBAR with the traditional type of FBAR, we observed a larger change in the resonant frequency downshift with the increase of light intensity. From Figure 5, when the UV intensity increases from 0.5 mW/cm^2^ to 2 mW/cm^2^, the frequency shift of the traditional FBAR increases from 15 kHz to 80 kHz, while the frequency shift of the proposed FBAR increases from 60 kHz to 250 kHz.

The frequency downshifts in both types of the devices have positive linear correlations with the UV light intensity, but different slopes which impacts the UV detector sensitivity. The new FBAR has a much larger frequency shift than that of the traditional FBAR at the same UV intensities from 0.5 mW/cm^2^ to 2 mW/cm^2^. Therefore, the proposed new FBAR structure which combines a traditional FBAR with a Au/n-ZnO Schottky diode has a much higher sensitivity than the traditional FBAR, verifying our device design proposal. The reasons for the different sensitivities are explained next.

## Discussion

The semiconductor ZnO has a direct band gap of 3.37 eV, which corresponds to UV light's energy. Therefore, pairs of holes and electrons will be generated when UV light illuminates the ZnO layer. However, the photo-generated holes recombine with the surface adsorbed oxygen ions. This results in free carriers due to the excess electrons^19^,^20^. The photo-generated carriers are confined in the surface layer of ZnO, resulting in an increase in the surface sheet conductivity, *σ*_s_. The relationship between the surface conductivity and acoustic wave velocity, *ν*, in the ZnO surface layer is given in the equation below^21^,^22^,^23^,^24^,


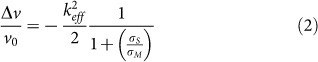
where *v*_0_, Δ*v*, 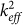, *σ_S_* and *σ_M_* are the intrinsic acoustic velocity, variation of acoustic velocity, electromechanical coupling coefficient of the FBAR device, surface conductivity of ZnO and bulk conductivity of ZnO below the surface layer, respectively.

**Figure 6:**
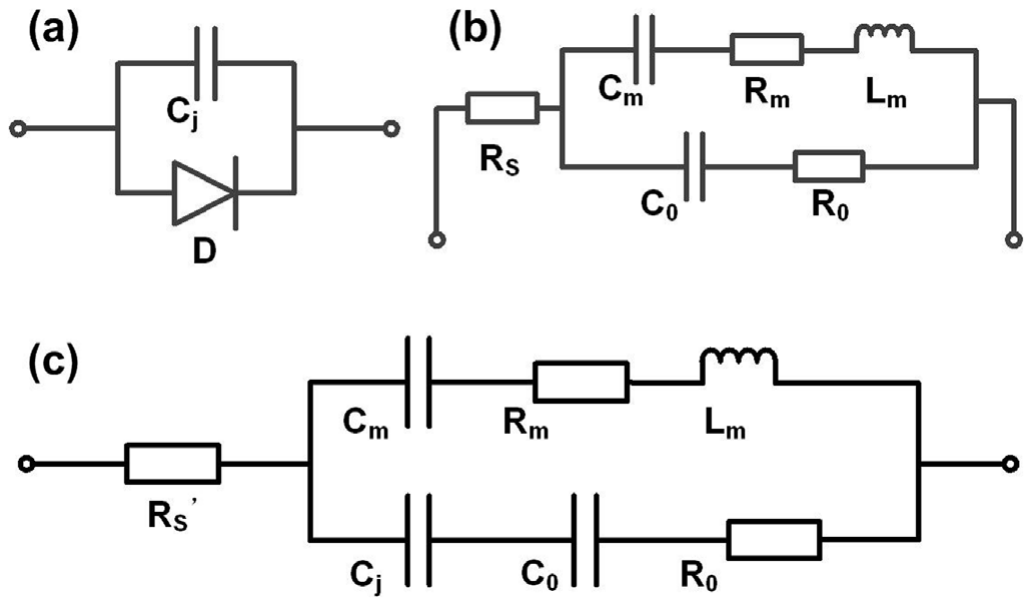
Equivalent circuit models of (a) a Schottky diode; (b) a FBAR device; and (c) a FBAR device with an n-ZnO semiconductor layer.

When the surface sheet conductivity increases, the acoustic velocity decreases. Equation (3) shows that the resonant (or fundamental) frequency *f_0_* is determined by the acoustic velocity *v* and the thickness of the piezoelectric layer *t_p_*. Under UV light exposure, the acoustic velocity decreases. This in-turn, causes the frequency to decrease as determined by the following equation,


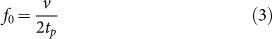
The mechanism for the much-improved performance of UV light sensing for the newly proposed FBAR can be explained by the equivalent circuit of the device. As shown in Figure 1b, a Schottky diode is stacked on the FBAR's piezoelectric layer. The capacitance of the Schottky barrier is in series with the static capacitance, *C*_0_, of the FBAR. The equivalent circuit of a Schottky diode and a FBAR device is shown in Figure 6. Specifically, Figure 6a is the equivalent circuit of the Schottky diode, and Figure 6b is the modified Butterworth-Van Dyke (MBVD) model of a typical FBAR device^25^. According to the structure, the model of the FBAR integrated with an n-ZnO layer can be obtained by combining the two models, as shown in Figure 6c.

In the MBVD equivalent circuit, R_0_, C_m_, L_m_, R_m_, and R_s_, are the FBAR's resistance due to medium loss, dynamic capacitance, dynamic inductance, resistance due to mechanical loss, and lead series resistance, respectively. The conventional MBVD model parameters can be extracted from measurement results^25^.

The Schottky depletion width, X_d_, and Schottky diode capacitance, C_j_, can be expressed as:


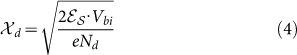

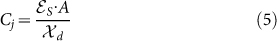


where *ε_S_*, *V_bi_*, *A*, *e* and *N_d_* are the dielectric constant of ZnO, built-in potential, FBAR's active area, electron charge, and doping concentration, respectively. When the Schottky junction is irradiated by UV light, the barrier height of the Schottky junction will decrease due to the excess carriers generated in the depletion region, resulting in a decrease of the built-in potential, *V_bi_*, in equation(4). These relationships show that the frequency shift under different UV intensities can be simulated from the equivalent circuit model. Here, the Advanced Design System (ADS) circuit simulation software was used to model the device.

**Figure 7:**
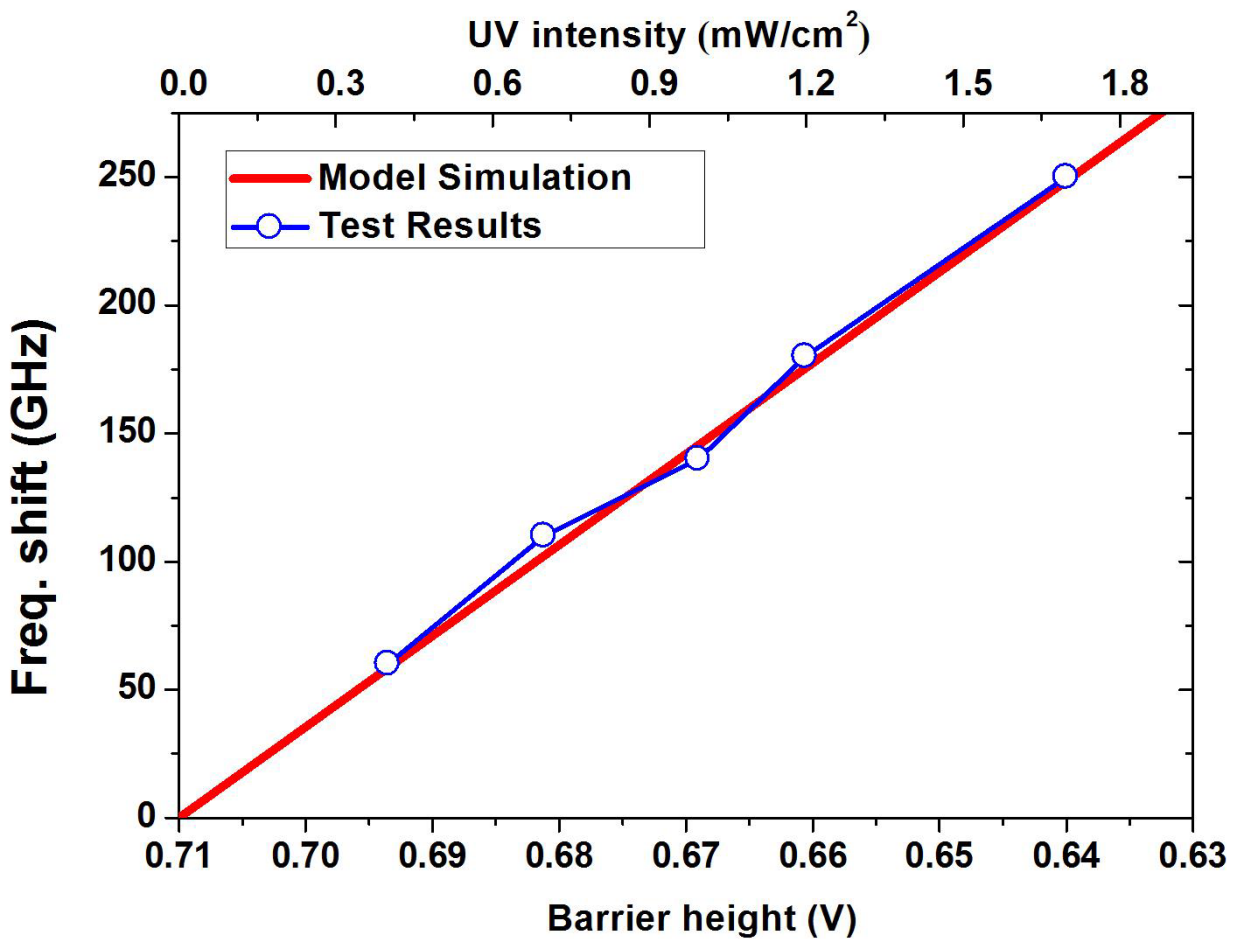
Frequency shift as a function of Schottky barrier height obtained by simulation and comparison with the experimental results.

The simulation results show that both the barrier height and the depletion width decreases under UV light exposure. Correspondingly, the Schottky diode capacitance increases, resulting in downshift of the resonant frequency. The numerical relationship between barrier height and frequency shift is shown in Figure 7 (top and bottom x-axes). When the UV intensity becomes larger, the barrier height decreases. When the barrier height of the Au/ZnO diode decreases from 0.71 V to 0.64 V, the resonant frequency downshift is from 0 to 250 kHz, in good agreement with the experiments.

Table 1 shows the effect of temperature on UV sensing based on the proposed FBAR device. As temperature increases, the resonant frequency shift, Δf_r_, under UV light decreases. For example, at 105°C, the frequency shift is only 13 kHz, about one fifth of its value at 30°C. As mentioned above, the new type of FBAR has a smaller TCF than that of the traditional FBAR. This improvement of TCF can be explained by the circuit model. For simplicity, we may consider the 56 kHz frequency shift on temperature variation from 30°C to 105°C is mainly caused by the introduction of the n-ZnO layer into the new FBAR, then for the same temperature variation, it will change the FBAR capacitance, C_0_, which, in-turn, will cause more than 3 MHz frequency shift, much larger than that induced by the change of C_j_. Thus C_j_ can be treated as constant when temperature changes. Since C_0_ and C_j_ are in series, the total capacitance, C, is as follow,


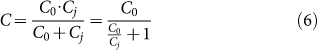
which is smaller than *C*_0_. Therefore, the change induced by temperature would be smaller for the total capacitance as well. In other words, the variation of *C*_0_ caused by temperature change is weakened by the introduction of the Schottky diode, leading to smaller TCF for the new FBARs compared to that of the traditional FBARs. This is a desirable property for electronic applications. Alternatively, the relationship between the frequency shift Δf_r_ and temperature can be utilized to develop the FBAR temperature sensor. For this example, a temperature sensitivity of ~ 0.75 kHz/°C can be obtained for the new type of FBARs.

In summary, we proposed a new structure FBAR device which incorporates an n-type ZnO layer between the top electrode and ZnO piezoelectric layer, and forms a Schottky diode. The Schottky diode makes a positive contribution to FBAR's response to UV light. UV sensing performance of both the traditional FBAR and the newly proposed FBAR were investigated and discussed. The frequency response of the new structure FBAR is much larger than that of the traditional FBAR. Moreover, an equivalent circuit model, namely FBAR's MBVD model in series with a Schottky diode, was presented. This model was used to explain the improved performance of the new device.

## Methods

### Device structure

The traditional FBAR devices were fabricated on 500 μm thick silicon wafers. First, the 100 nm thick Al layer bottom electrodes, were deposited by DC magnetron sputtering, and were then patterned with photolithography and a lift-off process. Next, a 2 μm ZnO piezoelectric layer was deposited by reactive magnetron sputtering^26^. Then vias through the ZnO layer to connect to the bottom electrodes were formed by patterning and etching the ZnO layer. Next, the top electrode, a 60 nm thick Au layer deposited by E-beam evaporation, was formed by photolithography and a lift-off process. Then, the bulk silicon under the FBAR was removed by deep reactive ion etching (DRIE, Oxford Instruments, Plasmalab System 100). The size of the active area of the FBAR is 200 × 200 μm^2^. The ZnO layer was characterized by XRD (Empyrean Panalytical) and SEM (Hitachi S-4800). For the new type of FBAR devices we proposed, an n-type ZnO semiconductor layer which was deposited by atomic layer deposition (ALD, Kurt J. Lesker Company, ALD-150LX) was introduced between the ZnO piezoelectric layer and the top Au electrode.

### UV sensing

A UV curing light whose intensity could be adjusted was used as a UV light source. A 365 nm optical filter was attached to restrict the wavelength of UV light. The frequency response was investigated at various UV intensities using a network analyzer (E5071C, Agilent), which was controlled by a LabView-based software through general purpose interface bus (GPIB).

## Author Contributions

X.B. deposited ZnO, Au and Al thin films, etched the silicon substrate, fabricated FBAR devices; X.B. and G.C. conducted the characterization of the ZnO materials. X.B. set up the UV sensing experiment and G.C. assisted the sensing experiments. H.J., X.W., S.D., J.K.L., M.J.D. and B.Q. supervised the project, analyzed the results, and contributed to writing the manuscript. All authors contributed to the interpretations and discussions of the research work.

## Figures and Tables

**Table 1:** Temperature effect on UV sensing performance of the new FBAR device under a UV light an intensity of 0.4 mW/cm^2^

Temperature (°C)	30	55	80	105
Δf_r_ (kHz)	69	51	21	13
